# Die Umsetzung einer adaptiven multimodalen interdisziplinären Schmerztherapie am Beispiel einer MVZ-Struktur aus Dresden

**DOI:** 10.1007/s00482-026-00936-7

**Published:** 2026-04-01

**Authors:** B. Schönbach, T. Kupke, U. Ludwig, R. Scharnagel, R. Sabatowski

**Affiliations:** 1 UniversitätsSchmerzCentrum, Universitätsklinik und Medizinische Fakultät „Carl Gustav Carus“, Technische Universität Dresden, Fetscherstr. 74, 01307 Dresden, Deutschland; 2 Zentrum für ganzheitliche Schmerztherapie Dresden MVZ GmbH, Dresden, Deutschland; 3 Klinik für Anästhesiologie und Intensivtherapie, Universitätsklinik und Medizinische Fakultät „Carl Gustav Carus“, Technische Universität Dresden, Dresden, Deutschland

**Keywords:** Chronischer Schmerz, Interdisziplinäre multimodale Schmerztherapie, Assessment, Chronifizierung, Ambulante Schmerztherapie, Chronic pain, Interdisciplinary multimodal pain therapy, Assessment, Chronification, Outpatient pain therapy

## Abstract

Chronische Schmerzen sind durch hohe und steigende Prävalenzen gekennzeichnet und gehen mit erheblichen funktionellen Beeinträchtigungen sowie erheblichen gesundheitsökonomischen Kosten einher. Qualitative und quantitative Versorgungsdefizite äußern sich in Unzufriedenheit der Behandelten, erhöhten Folgekosten und der Begünstigung weiterer Chronifizierungsprozesse. Die interdisziplinäre multimodale Schmerztherapie berücksichtigt das zugrunde liegende biopsychosoziale Bedingungsgefüge chronischer Schmerzerkrankungen und gilt als evidenzbasierter Goldstandard der Behandlung. Entsprechende Therapiekonzepte sind zeit- und personalintensiv und stehen in der Regelversorgung überwiegend im (teil-)stationären Setting zur Verfügung. Für den ambulanten Bereich bedarf es daher geeigneter Versorgungsstrukturen, die sich am Konzept der interdisziplinären multimodalen Schmerztherapie orientieren und eine zielgerichtete Behandlung ermöglichen. Bislang wurden solche Ansätze vor allem im Rahmen von Modellprojekten und Selektivverträgen umgesetzt. Wesentliche Hürden bestehen insbesondere in unzureichenden Vergütungs- und Abrechnungsmöglichkeiten innerhalb des einheitlichen Bewertungsmaßstabs, der die interdisziplinäre Zusammenarbeit nicht adäquat abbildet. Am Beispiel einer Struktur, die einem Medizinischen Versorgungszentrum (MVZ) ähnlich ist, wird das Modell einer adaptiven multimodalen interdisziplinären Schmerztherapie vorgestellt. Dieses trägt den komplexen Behandlungsanforderungen chronischer Schmerzpatient*innen Rechnung, ermöglicht einen bedarfsgerechten Einsatz personeller Ressourcen und fördert die strukturierte Zusammenarbeit der beteiligten Fachdisziplinen. Zentrales Element ist ein sich über Feedbackschleifen kontinuierlich optimierender diagnostischer und therapeutischer Prozess, in dem multimodale Therapieelemente interdisziplinär koordiniert und über gemeinsam einsehbare digitale Akten sowie Fallbesprechungen verknüpft werden.

## Abstract (en)

Chronic pain is characterized by high and increasing prevalences and is associated with significant functional impairments and substantial healthcare costs. Qualitative and quantitative gaps in patient care are manifested as patient dissatisfaction, increased follow-up costs and the promotion of further chronification processes. Interdisciplinary multimodal pain therapy considers the underlying biopsychosocial factors of chronic pain conditions and is considered the evidence-based gold standard of treatment. Corresponding treatment approaches are time-consuming and personnel-intensive and are predominantly available in (partial) inpatient settings within standard care. Therefore, suitable care structures are needed for outpatient care that are based on the concept of interdisciplinary multimodal pain therapy and enable targeted treatment. To date, such approaches have primarily been implemented within the framework of pilot projects and selective contracts. Significant obstacles include inadequate reimbursement and billing options within the standardized fee schedule, which do not adequately reflect interdisciplinary collaboration. Using the example of a medical care center (MVZ) structure, a model for adaptive, multimodal, interdisciplinary pain therapy is presented. This model addresses the complex treatment requirements of chronic pain patients, enables the needs-based allocation of personnel resources and promotes structured collaboration among the participating medical disciplines. A central element is a diagnostic and therapeutic process that is continuously optimized through feedback loops, in which multimodal therapy elements are coordinated across disciplines and linked via shared digital files and case discussions.

## Hintergrund

Repräsentative Erhebungen in Deutschland dokumentieren, dass bis zu 30 % der Menschen chronische, nichttumorbedingte Schmerzen angeben, bei ca. 2,3 % liegt eine Schmerzkrankheit, gekennzeichnet durch schmerzbedingte körperliche, psychische und soziale Beeinträchtigungen, vor [16, 17, 35]. Daten von gesetzlichen Krankenkassen zeigen zudem eine stetige Zunahme der schmerzbezogenen Diagnosezahlen; diese Daten werden auch aus unterschiedlichen Erhebungen anderer Länder berichtet [12, 14, 15, 32, 35]. Darüber hinaus ergeben sich aus diagnostischen und therapeutischen Interventionen, Arbeitszeitausfällen und Frühberentungen erhebliche ökonomische Folgekosten [33, 34]. Unterschiede in der Krankheitsausprägung können dazu führen, dass die entsprechenden Patient*innen mit unterschiedlichen Intensitäten in verschiedenen Sektoren (ambulant bis stationär) behandelt werden sollten [26].

Chronische Schmerzen sind als eigenständige komplexe Krankheit zu verstehen. Dem wurde im Jahr 2009 mit der Einführung der Diagnose F45.41 in die ICD-10 Rechnung getragen. Das biopsychosoziale Modell bietet hier eine grundlegende Perspektive zur Genese und Behandlung [29]. Vor diesem Hintergrund hat sich die interdisziplinäre multimodale Schmerztherapie (IMST) als Goldstandard in der Behandlung von Patient*innen mit fortgeschrittener Schmerzchronifizierung etabliert [2, 3, 5, 22]. Zielstellungen, Inhalte und Strukturmerkmale der Therapie bzw. Einrichtungen wurden von einer Ad-hoc-Kommission der Deutschen Schmerzgesellschaft e. V. herausgearbeitet [1–4, 24, 26]. *Multimodal *kommen ärztliche, physio- und psychotherapeutische Komponenten in *interdisziplinärer Zusammenarbeit* zum Einsatz [2, 3]. In einem regelhaft vorgeschalteten interdisziplinären multimodalen Assessment (IMA) erfolgen die Indikationsprüfung sowie die Festlegung individueller Therapieziele [6]. Die Behandlungsform der IMST wird bisher überwiegend teil- oder vollstationär angeboten, wobei sich Rahmenbedingungen, wie Behandlungsintensität, Inhalte und strukturelle Vorgaben an den Vorgaben des aktuellen Operationen- und Prozedurenschlüssels orientieren [7].

Die IMST als Behandlungsansatz einer komplexen Erkrankung bedarf einer engen zeitlichen und inhaltlichen Abstimmung der verschiedenen beteiligten Berufsgruppen. Hierfür müssen ausreichend Möglichkeiten zur Abstimmung der interdisziplinären Zusammenarbeit vorgehalten werden, um jederzeit einen seitens der Strukturvorgaben geforderten integrativen Behandlungsansatz zu gewährleisten. Dabei finden im Sinne eines iterativen Prozesses gemeinsame Teambesprechungen statt, in denen die Relevanz erhobener Befunde diskutiert, das Behandlungsvorgehen miteinander abgestimmt, die Zielerreichung kontrolliert und Veränderungen im Vorgehen abgeleitet werden. Dieser Prozess kann durchaus auch als Strategie interpretiert werden, in der nicht nur die Versorgung der Patient*innen verbessert wird, sondern darüber hinaus auch die professionelle Praxis und damit auch die Qualität der erbrachten Leistung angepasst und optimiert werden [30].

Die ambulante Behandlung von Menschen mit chronischen Schmerzen weist in Deutschland quantitativ und qualitativ erhebliche Defizite auf. Ergebnisse einer repräsentativen Stichprobe der deutschen Bevölkerung zeigen, dass nur etwa ein Drittel der Personen mit beeinträchtigenden Schmerzen in schmerztherapeutischer Behandlung ist. Zudem war von diesen Personen ein Drittel mit der schmerztherapeutischen Behandlung unzufrieden oder sehr unzufrieden [16]. Patient*innen warten je nach Bundesland 2 bis 5 Jahre auf eine qualifizierte schmerzmedizinische Behandlung [6].

Die Versorgung im ambulanten Sektor ist bei Patient*innen mit chronischem Schmerz oftmals gekennzeichnet von einer Überdiagnostik auf somatischer Ebene, während die vielfältigen psychosozialen Faktoren der Schmerzchronifizierung kaum Berücksichtigung finden [11]. Dem gegenüber stehen Leitlinienempfehlungen, z. B. die der Nationalen VersorgungsLeitlinie Nicht-spezifischer Kreuzschmerz. Demnach sollten nach Ausschluss sogenannter „red flags“ frühzeitig bereits nach 6 Wochen Schmerzdauer psychosoziale Risikofaktoren für die Chronifizierung identifiziert und therapeutisch adressiert werden [5].

Auch wenn ambulante Behandlungen inzwischen durch ein höheres Maß an Multidisziplinarität gekennzeichnet sind, fehlt häufig eine teamintegrierte interdisziplinäre Zusammenarbeit der Fachgruppen, die eine Abstimmung der Behandlungselemente ermöglicht [19].

Die Ad-hoc-Kommission „IMST“ beschrieb verschiedene Problemfelder auf organisatorischer, Vergütungs- und qualifikationsbezogener Ebene, die die Umsetzung der Therapieform im ambulanten Bereich erschweren [26]. Die interdisziplinäre Behandlung erfordert eine enge Zusammenarbeit verschiedener Fachdisziplinen und geht mit einem erheblichen Ressourceneinsatz einher. Anforderungen an die Qualifikation des Personals sind hoch und umfassen für eine zweckmäßige Behandlung Betroffener u. a. spezielle Zusatzqualifikationen. Wesentlich sind zudem fehlende Vergütungs- und Abrechnungsmöglichkeiten des einheitlichen Bewertungsmaßstabs (EBM), der die komplexen Prozessanforderungen, insbesondere die Notwendigkeit eines strukturierten Austauschs der Fachdisziplinen, nicht abbildet [26].

Die Ad-hoc-Kommission hat sektorenübergreifende Strukturvorschläge für die IMST erarbeitet. Darin wird vorgeschlagen, dass im ambulanten Bereich Patient*innen mit geringerer Schmerzchronifizierung möglichst niedrigschwellig und berufsbegleitend behandelt werden sollten, um ein Fortschreiten der Chronifizierung zu verhindern [26]. Empfohlen wird zudem bei drohender und bereits fortgeschrittener Chronifizierung die Durchführung eines IMA [8]. Das IMA ermöglicht die umfassende Diagnostik chronischer Schmerzerkrankungen und kann aufgrund der ergebnisoffenen Ausrichtung auch als Steuerungsinstrument für die Weiterbehandlung hilfreich sein [26].

Diese o. g. schmerztherapeutischen Leistungen können durchaus in unterschiedlichen ambulanten Struktureinheiten erbracht werden. Das können einerseits sogenannte „Zentren für interdisziplinäre Schmerzmedizin“ sein, andererseits Praxen, die neben einer gebietsbezogenen Versorgungsstruktur spezialisierte Schmerztherapie oder einen zukünftig möglicherweise zu etablierenden Fachkundenachweis vorweisen. Entsprechend zu erfüllende Struktur- und Prozessparameter wurden erstmalig bereits 2011 beschrieben und im Wesentlichen 2016 von einer interdisziplinären Arbeitsgruppe bestätigt [25, 28].

Angebote des IMA oder der IMST im ambulanten Bereich wurden jedoch bislang vorrangig als Modellprojekte oder über Selektivverträge realisiert [26].

Dabei beziehen sich wissenschaftliche Erhebungen insbesondere auf den Bereich der Behandlung von Personen mit einer frühen Schmerzchronifizierung [20]. Darin zeigte sich, dass frühzeitig durchgeführte interdisziplinäre multimodale Assessments im Rahmen des Innovationsfondsprojekts „PAIN2020“ Verbesserungen in schmerzbezogenen, funktionellen und psychischen Parametern erzielen konnten. Diese waren allerdings unabhängig davon, ob Assessments rein regelversorgungsbasiert medizinisch oder interdisziplinär im Rahmen der neuen Versorgungsform umgesetzt wurden [31].

Es wäre jedoch ein Fehler, hinsichtlich der ambulanten Versorgung von Patient*innen mit Schmerzen ausschließlich die Gruppe mit einer frühen oder niedriggradigen Chronifizierung zu adressieren. Patient*innen mit einer fortgeschrittenen Schmerzchronifizierung benötigen ebenfalls adäquate interdisziplinäre ambulante Behandlungsstrukturen, zumal der Zugang zu spezialisierten Behandlungen der IMST im (teil-)stationären Sektor oftmals nicht oder nur mit erheblicher zeitlicher Verzögerung möglich bzw. aufgrund von individuellen Hinderungsgründen (z. B. berufliche Situation, Kinderbetreuung) nicht umsetzbar ist. Darüber hinaus ist zur Absicherung der Therapieergebnisse sowie zur Unterstützung des Alltagstransfers der Behandlungsempfehlungen nach (teil-)stationärer Behandlung oftmals eine längerfristige schmerzmedizinische Nachbetreuung notwendig. Für diese Gruppe müssen daher zielgerichtete ambulante Behandlungskonzepte vorgehalten werden, die Interdisziplinarität und Multimodalität als Kernelemente des Behandlungsvorgehens beinhalten.

Zusammengefasst lassen sich die Herausforderungen und die Notwendigkeit einer adäquaten ambulanten schmerzmedizinischen Diagnostik und Behandlung folgendermaßen beschreiben: Aufgrund der demografischen Entwicklung sowie der Zunahme von Schmerzerkrankungen in der Bevölkerung und den oftmals unzureichenden Versorgungsangeboten und der daraus resultierenden Unterversorgung sind eine auch quantitative Erweiterung und ein innovativer Ausbau ambulanter Strukturen essenziell [6, 10, 14, 15, 17, 26]. Zu den im ambulanten Setting umzusetzenden Aufgaben zählen die Abkehr von rein unimodalen oder nicht teamintegrierten Diagnostik- und Behandlungsansätzen, die oftmals von fehlender oder unzureichender interdisziplinärer Kommunikation gekennzeichnet sind. Auch kommt dem ambulanten Sektor eine große Bedeutung in der Fallsteuerung, idealerweise durch frühzeitiges IMA, sowie in der langfristigen Betreuung zu.

Dem entgegen steht aber bislang, dass solche Modelle in der Regelversorgung nicht vorgesehen sind. Auch im gegenwärtig in Überarbeitung befindlichen „DMP Chronischer Rückenschmerz“ ließ sich in der bisherigen Version keine Struktur- bzw. Vergütungsempfehlung für ein IMA durchsetzen [13].

## Adaptive ambulante interdisziplinär-multimodale Schmerztherapie

Das *Zentrum für Ganzheitliche Schmerzmedizin* in Dresden setzt in der Struktur eines medizinischen Versorgungszentrums (MVZ) seit 2017 eine adaptive und interdisziplinäre ambulante Behandlung von Patient*innen mit chronischen Schmerzerkrankungen um. Kernelemente der Struktur und des Behandlungsvorgehens umfassen folgende Bestandteile.

**Abb. 1:**
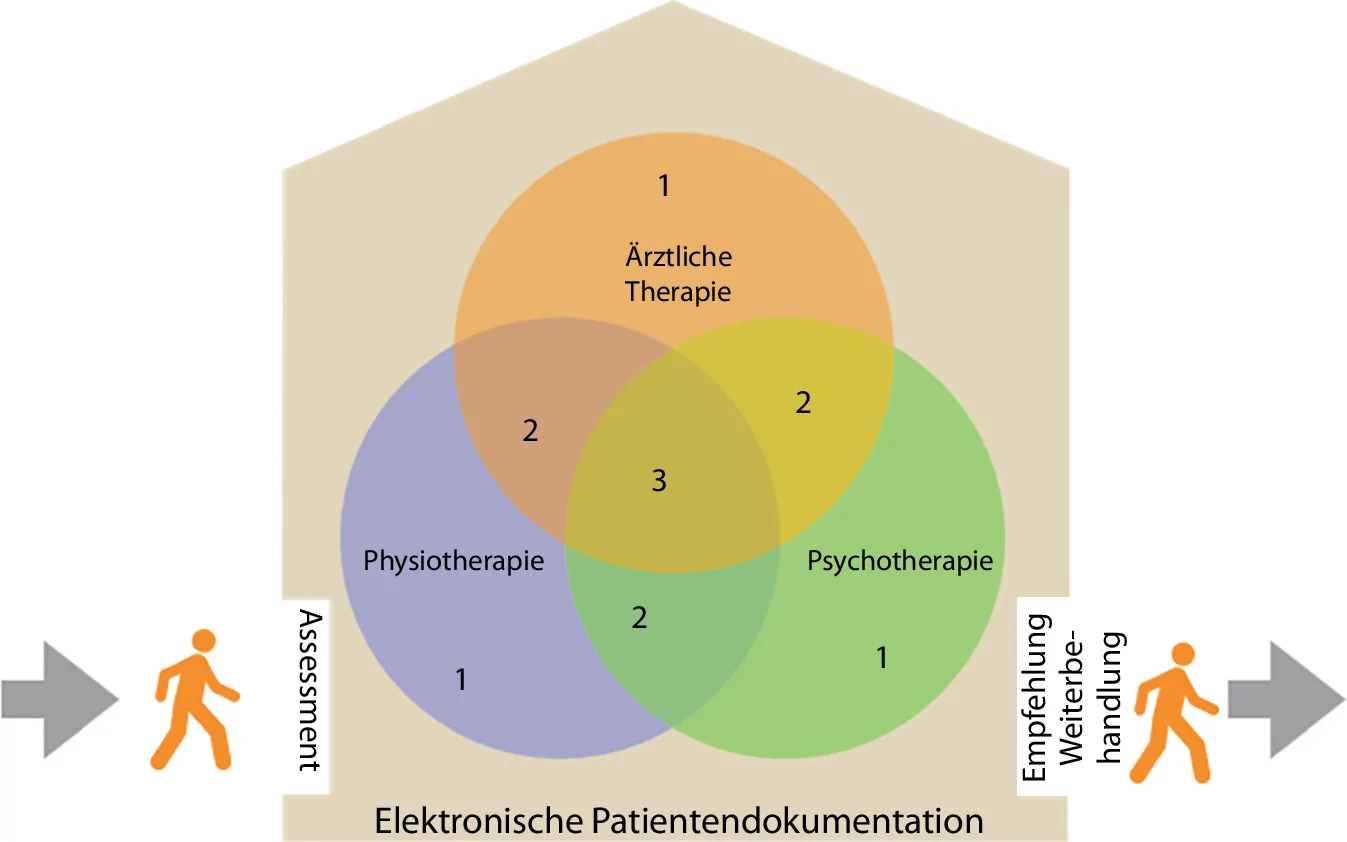
Organisatorischer und fachlicher Rahmen. *1:* Monodisziplinäre Behandlung, *2*: abgestimmte Behandlung durch 2 Fachdisziplinen, *3*: abgestimmte Behandlung durch 3 Fachdisziplinen

Den Mittelpunkt der Struktur (Abb. 1) bildet ein multiprofessionelles, interdisziplinäres Team, bestehend aus 8 Fachärzt*innen mit der Zusatzbezeichnung „Spezielle Schmerztherapie“, 6 psychologischen Psychotherapeut*innen und 5 Physiotherapeut*innen. Diese werden durch weitere Fachkräfte unterstützt. Hierzu zählen 11 algesiologische Fachassistentinnen, eine Sozialberaterin sowie eine Osteopathin. Jede Berufsgruppe ist eigenständig mit definierten Verantwortlichkeiten und Zielstellungen organisiert. Übergeordnet besteht ein Konsens bezüglich gemeinsamer Therapieziele, die sich u. a. an den bereits von Arnold et al. definierten Therapiezielen der IMST orientieren und für alle Teammitglieder bindend sind [2]. Den organisatorischen Rahmen bilden eine fachärztlich geleitete Managementstruktur und ein Verwaltungsteam in einer MVZ-Struktur. Hierfür ist es entscheidend für die Realisierung der interdisziplinären Struktur, dass die erforderlichen vertragsärztlichen und psychotherapeutischen Sitze im jeweiligen Planungsbereich vorhanden sind und in das MVZ eingebracht werden können. Außerdem bedarf es einer Zulassung für die Physiotherapie. Im MVZ vereint sind 8 ärztliche und 3 psychotherapeutische Sitze sowie die physiotherapeutische Zulassung. Damit werden die Voraussetzungen zur Erbringung aller Leistungen erfüllt. Mit dieser MVZ-Struktur ist es auch möglich, medizinisches Fachpersonal von organisatorischen und administrativen Aufgaben zu entlasten und somit mehr Behandlungskapazität zur Verfügung zu stellen. Die fachliche Weisungsbefugnis liegt auf ärztlicher Seite, sodass auf der Basis eines gemeinsam erarbeiteten Grundkonzepts jederzeit ein dem aktuellen medizinischen Standard folgendes Vorgehen gewährleistet werden kann.

Zu den Besonderheiten dieser Struktur gehört die gemeinsam genutzte elektronische Dokumentationsplattform. Jedes Team ist über diese umfangreiche patient*innenbezogene elektronische Kommunikationsstruktur mit den anderen Behandlungsteams verbunden. Innerhalb der elektronischen Patientenakte erfolgt die gesamte Dokumentation der Befunde und Verläufe, sodass sich alle Teams jederzeit über den aktuellen Stand der Behandlung informieren können und somit Informationslücken vermieden werden. Inhalt dieser Dokumentation sind auch die Ergebnisse unterschiedlicher fachspezifischer Assessments sowie der verschiedenen Teambesprechungen.

Ein weiterer wesentlicher Aspekt der Organisationsstruktur ist in den strukturierten Fallbesprechungen zu sehen. Neben der im Rahmen der Qualitätssicherungsvereinbarung von der Kassenärztlichen Vereinigung (KV) geforderten monatlichen Schmerzkonferenz finden wöchentliche Fallbesprechungen in einem sogenannten „Marktplatzsystem“ statt. Im Vorfeld dieser Besprechung definiert jede*r primär zuständige Behandler*in, welche Mitbehandelnden aus einem anderen Team für den zu diskutierenden Fall konsultiert werden sollen, und terminiert ein spezifisches Treffen innerhalb des dafür vorgehaltenen Zeitfensters. Das bedeutet, dass innerhalb dieses „Marktplatzsystems“ zahlreiche Treffen kleiner Subteams, typischerweise bestehend aus zwei bis drei für den Fall relevanten Behandelnden, stattfinden. Diese Struktur gewährleistet, dass ausschließlich die tatsächlich in den jeweiligen Fall involvierten Personen effizient gebunden sind. Somit können gleichzeitig durchschnittlich 5 bis 10 Patient*innen pro Behandler*in und Woche in diesem Format parallel diskutiert und die weitere Behandlung miteinander abgestimmt werden.

**Abb. 2:**
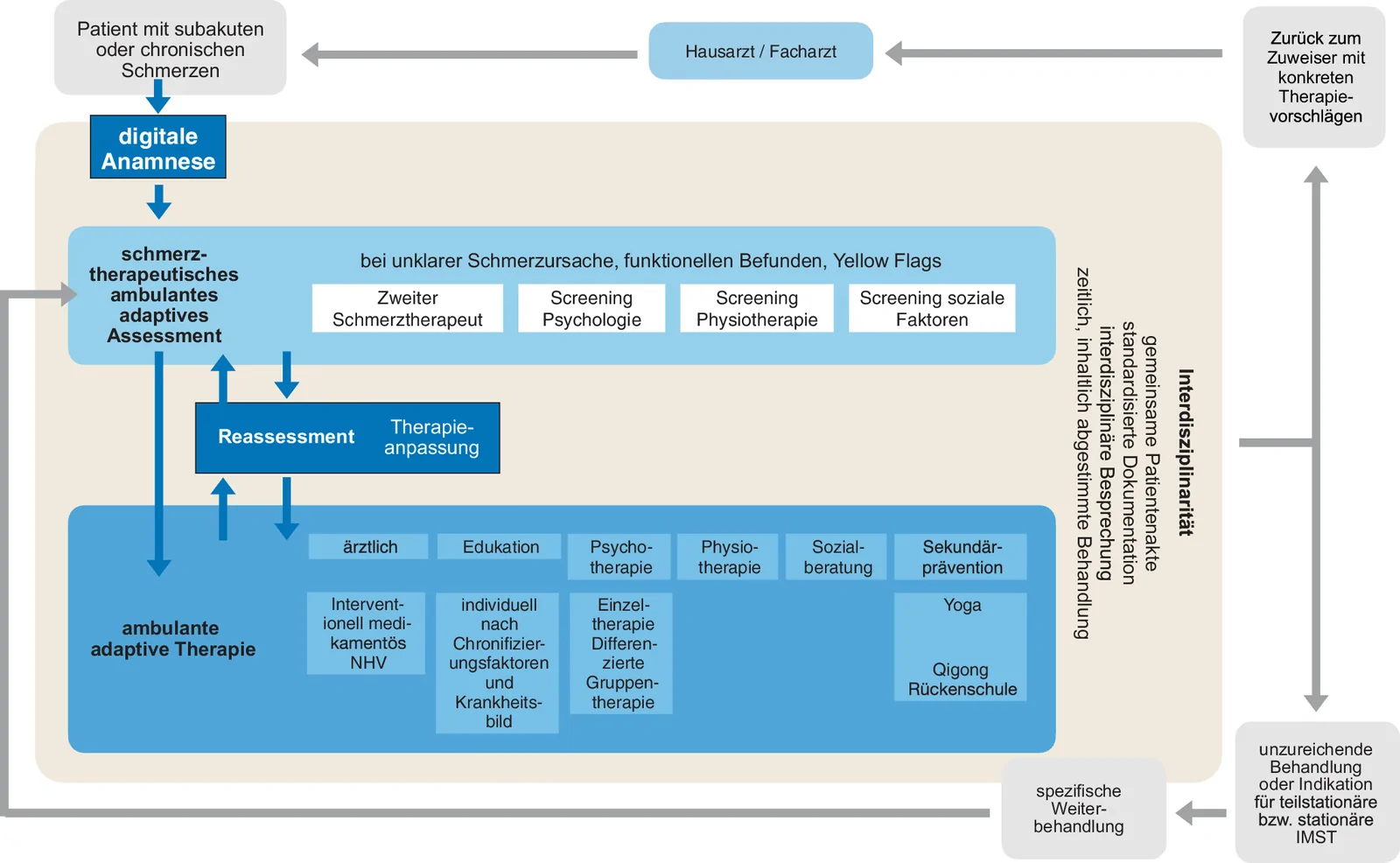
Adaptiver interdisziplinärer multimodaler Diagnostik- und Behandlungsprozess. *NHV* Naturheilverfahren

Als weitere Besonderheit wird der Diagnostik- und Behandlungsprozess als dynamischer, sich selbst über Feedbackschleifen rückkoppelnder und optimierender Prozess konzipiert (Abb. 2). Das Kernelement bilden das Eingangsassessment und Reassessment. Auf der Basis der Vorbefunde und des vor dem Erstkontakt auszufüllenden standardisierten Deutschen Schmerzfragebogens findet ein modifiziertes Eingangsassessment statt. D. h., primär erfolgt die Ersteinschätzung neuer Patient*innen durch eine erfahrene ärztliche Fachkraft für Schmerztherapie. Auf dieser Basis wird entschieden, ob und welche weiteren Fachdisziplinen einbezogen und Maßnahmen eingeleitet werden. Der Umfang der zusätzlich einbezogenen Disziplinen wird primär im Rahmen des Eingangsassessments festgelegt, kann aber bei neuen Erkenntnissen im Rahmen der primär hinzugezogenen Disziplinen durchaus auch erweitert werden. Die Ergebnisse des Assessments werden in der gemeinsamen digitalen Akte dokumentiert. Komplexe Fälle werden zeitnah in den interdisziplinären Fallbesprechungen unter Teilnahme aller am Zentrum vorhandenen Fachdisziplinen bewertet. Das Ergebnis ist ein abgestimmter interdisziplinärer Behandlungsplan, der die Grundlage der weiteren Therapie bildet. In der Behandlung werden die wesentlichen Ursachen und aufrechterhaltenden Bedingungen adressiert, um vorhandene Ressourcen möglichst effizient und adaptiv einsetzen zu können. Diese Priorisierung einer Hauptleistung führt zu unterschiedlichen Behandlungsintensitäten. Bei der Priorisierung psychologischer Faktoren beträgt die Therapiedauer bei einer Sitzung pro Woche insgesamt 8 Wochen. Bei einem Schwerpunkt Physiotherapie werden in diesem Zeitrahmen 1–2 Einheiten pro Woche durchgeführt. In ca. 17 % der Fälle wird eine sogenannte „mehrfach modale“ Behandlung (z. B. nur 2 Fachbereiche, vgl. Abb. 1) durchgeführt. Patienten mit chronischen Schmerzen und „yellow flags“ erhalten in der Regel eine Behandlung durch 2 Fachbereiche; besteht bereits ein hoch chronifiziertes Schmerzsyndrom, so sind in der Regel alle 3 Fachdisziplinen in die Behandlung eingebunden. Eine im klassischen Sinne interdisziplinär multimodale Behandlung mit gleichzeitiger Inanspruchnahme aller Fachdisziplinen ist demgegenüber nur für einen deutlich geringeren Anteil (ca. 4–5 %), in der Regel hochkomplexe Fälle, erforderlich. Besteht bei hoch chronifizierten Schmerzen eine Indikation für eine (teil-)stationäre Behandlung erfolgt entweder direkt eine entsprechende Überweisung oder diese erfolgt im Therapieverlauf nach entsprechendem Reassessment (ca. 5 % der Fälle/Jahr). Nach Abschluss eines ersten Behandlungsabschnitts erfolgt standardisiert ein ärztlich-schmerztherapeutisches Reassessment, gefolgt von einer erneuten interdisziplinären Besprechung mit der Option zur Anpassung des Behandlungsplans bzw. der beteiligten Teamzusammensetzung. Diese Reassessments und die daraus resultierenden Behandlungsschritte können im Verlauf durchaus mehrfach durchgeführt werden. Auch wenn die vorhergehende Behandlung im Team bestehend aus Psycho- und Physiotherapie durchgeführt worden ist, bleibt unabdingbar, dass auch in dieser Konstellation, d. h. mit vorhergehendem Therapieabschnitt ohne regelmäßige ärztliche Kontakte, das Reassessment mit ärztlicher Beteiligung stattfindet. Damit bleibt die ärztliche Kontrolle des Behandlungsprozesses jederzeit gewährleistet. Unabhängig von den individuellen Behandlungsabläufen werden gruppenbasierte edukative Angebote bzw. übende Verfahren vermittelt. Dies beinhaltet auch Schulungsmaßnahmen zu verschiedenen für die Patient*innen relevanten Themengebieten (u. a. Chronifizierungsfaktoren, Kopfschmerz, Stress, Bewegung), die der Steigerung der Eigenkompetenz sowie Prävention einer Chronifizierung dienen. Der Umfang der Gruppentherapie reicht von 3–6 h (Basisgruppe) bis zu 15–33 h (verhaltenstherapeutische Gruppentherapie). Die Finanzierung erfolgt über die KV bei Vorliegen der Zusatzqualifikation „Gruppenpsychotherapie“. Nach erfolgreicher Therapie erfolgt die Rücküberweisung zur primären Zuweisung, wobei Behandlungsempfehlungen gegeben werden. Beim Vorliegen von „red flags“ oder (teil-)stationärer Behandlungsbedürftigkeit erfolgt eine externe Überweisung, wobei in der Regel die weitere schmerzspezifische Betreuung nach abgeschlossener (teil-)stationärer Behandlung zur Aufrechterhaltung der Therapieergebnisse innerhalb der zuvor beschriebenen Struktur gewährleistet werden kann.

Innerhalb dieser Struktureinheit können pro Jahr > 4000 Patient*innen mit chronischen Schmerzen unterschiedlicher Ausprägung (jeweils ca. 33 % MPSS 1–3) betreut werden. Hierbei verteilen sich die Hauptdiagnosen zu ca. 80 % auf Schmerzen des Stütz- und Bewegungssystems und zu jeweils weiteren 10 % auf Kopfschmerzen bzw. neuropathische Schmerzen. Pro Arzt bzw. Ärztin können 500 Patient*innen/Quartal betreut werden; die Anzahl der Neuaufnahmen liegt bei ca. 1100 Patient*innen/Jahr. Ca. 25 % der Patient*innen werden in der Folge psycho- und/oder physiotherapeutisch mitbehandelt. 20 % aller Behandelten werden in der interdisziplinären Schmerzkonferenz und im sogenannten „Marktplatz“ besprochen.

Die Struktur der adaptiven multimodalen interdisziplinären Ambulanz unterscheidet sich in wesentlichen Punkten von den bisher beschriebenen Klassifikationskriterien für ambulante Einrichtungen (s. Tab. 1; [25, 28]).

**Tab. 1:** Kriterienkatalog. (Adaptiert nach [25, 28])

	Ambulanz/Praxis	Adaptive interdisziplinäre multimodale Ambulanz
Struktur	Überwiegend Einzelpraxis	MVZ-ähnliche Struktur
Anzahl der Mitarbeitenden	i. d. R. 4–8	45
Behandlerteam aus unterschiedlichen ärztlichen Professionen	Nein	Ja*
Integration Psycho‑/Physiotherapie	Nein	Ja**
Ständige psychologische Leitung mit Zusatzqualifikation „Spez. Schmerzpsychotherapie“	Nein	Ja
IMA wird regelhaft durchgeführt	Nein	Ja
Ambulante IMST wird angeboten	Nein	Ja
Angebot für Prävention, Edukation und Gruppentherapie	Nein	Ja

*IMA* interdisziplinäres multimodales Assessment, *IMST* interdisziplinäre multimodale Schmerztherapie

*Fachärzt*innen für Allgemeinmedizin, Frauenheilkunde, physikalische und rehabilitative Medizin

**Davon 2 Personen mit Zusatzbezeichnung „Spezielle Schmerzpsychotherapie“

## Diskussion

Hohe und steigende Prävalenzen, Unterversorgung und Unzufriedenheit der Betroffenen verweisen auf die Notwendigkeit, die ambulante Versorgung von Menschen mit chronischen Schmerzen zu verbessern [10, 14–17]. Nicht zuletzt besteht diese auch vor dem Hintergrund der zunehmenden gesundheitspolitisch geforderten Ambulantisierung des Gesundheitswesens.

Bisherige Studien zu ambulanten Behandlungsansätzen in der Schmerzmedizin fokussierten im Wesentlichen auf frühe Chronifizierungsstadien und die Verhinderung weiterer Chronifizierung [20, 31]. Behandlungsansätze der IMST gelten als Goldstandard in der Behandlung von Patient*innen mit einer chronischen Schmerzerkrankung, sind jedoch in Deutschland fast ausschließlich teilstationär und stationär verfügbar [26]. Im ambulanten Rahmen führen unimodale, oft einseitig somatisch ausgerichtete Therapieangebote oder wenig verzahnte Behandlungen zu einer unzureichenden Besserung und tragen mitunter zur Chronifizierung bei [19].

Eine umfassende Diagnostik und zielgerichtete Steuerung der Fälle kann mithilfe des IMA gelingen, das jedoch nicht Bestandteil der Regelversorgung ist [8, 26]. Lediglich für Versicherte einzelner Krankenkassen gibt es im Rahmen eines Selektivvertrags mit der Deutschen Schmerzgesellschaft e. V. die Möglichkeit, ein IMA durchzuführen [9]. Erschwerend kommt hinzu, dass aufgrund oftmals fehlender etablierter Strukturen im ambulanten Sektor die (fachlichen) Voraussetzungen zur Durchführung eines IMA nicht gegeben sind [27]. Doch auch nach einem initialen Assessment und einer daraus resultierenden Behandlungsempfehlung bedarf es durchaus eines weiteren interdisziplinären Austauschs und der regelhaften Therapiekontrolle. Findet diese nicht statt, so zeigten Huge et al., dass die gewünschten Behandlungseffekte nicht erreicht werden konnten [18]. Bereits 2022 wurde von Kayser et al. ein Modellprojekt im Rahmen eines krankenkassenfinanzierten Selektivvertrags zur Entwicklung eines sektorenübergreifenden Behandlungskonzepts für Patient*innen mit chronischen Schmerzen entwickelt. Vorgeschlagen wurde eine 7‑stufige intersektorale Behandlung inklusive eines IMA als zentralem Steuerungselement [23]. Eingeschlossen waren u. a. ambulante, stationäre sowie telemedizinische Bereiche. Das hier dargestellte Modell der ambulanten adaptiven IMST vereint im Rahmen der Regelversorgung die auch im Modellprojekt von Kayser et al. vorgeschlagenen unterschiedlichen Bereiche unter einer übergeordneten Organisationsstruktur [23]. Hierbei werden die ärztlichen und psychotherapeutischen Leistungen über die entsprechende KV-Vergütung, die Physiotherapie als Einzelleistung nach dem Heilmittelkatalog abgerechnet. Organisatorische Aufgaben, die die MVZ-Struktur beinhaltet, werden nicht erstattet.

Im Rahmen eines primären Assessments empfohlene Behandlungen können in unterschiedlicher, auch wechselnder, therapeutischer Zusammensetzung und Intensität begleitet von regelhaften Reassessments in einer MVZ-ähnlichen Struktur angeboten und kontrolliert durchgeführt werden. Der Informationsaustausch und die Adressierung gemeinsamer Behandlungsziele werden einerseits durch eine von allen Disziplinen einsehbare und bearbeitbare digitale Patient*innenenakte und andererseits durch Teamsitzungen (gemeinsame interdisziplinäre Fallkonferenz, „Marktplatz“) gewährleistet. Eine Besonderheit gegenüber vielen (teil-)stationären Einrichtungen ist das adaptive Vorgehen. Die oft strengen Strukturvorgaben, die in vielen der Einrichtungen im (teil-)stationären Setting einerseits zu einer gleichbleibenden Behandlungsqualität führen sollen, führen auf der anderen Seite durchaus auch zu einer Einschränkung der Flexibilität. Dies bedeutet, dass in der (teil-)stationären Struktur, wie sie von der Ad-hoc-Kommission „IMST“ empfohlen wird, der Grad der Flexibilität, wie sie im dargestellten Modell angeboten wird, in der Regel nicht umsetzbar ist. Im engeren Sinne könnte man aufgrund des Umstands, dass teilweise im Behandlungsverlauf nur 2 verschiedene Disziplinen die Therapie durchführen, postulieren, dass dies der Definition der IMST widerspricht. Auf der anderen Seite bietet dieses adaptive Vorgehen aber deutlich mehr Flexibilität und darüber hinaus ermöglicht dieses Konzept auch, dass deutlich mehr Patient*innen effektiv und kontrolliert interdisziplinär multimodal behandelt werden können. Auch wird über dieses Modell gewährleistet, dass Patient*innen mit einem noch niedrigen Chronifizierungsgrad schmerztherapeutisch mitbehandelt werden können. Ob dieser frühe Zugang ein Fortschreiten einer Chronifizierung aufhalten kann, bleibt unklar. Die bisherigen Untersuchungen zu dieser Fragestellung konnten noch keine überzeugenden Ergebnisse erbringen [31]. So zeigte sich im Innovationsfondsprojekt „PAIN2020“ kein Unterschied in unterschiedlichen Outcomeparametern bei Patient*innen mit niedrigem Chronifizierungsgrad in Abhängigkeit davon, ob das primäre Assessment „nur“ durch eine schmerzmedizinisch erfahrene ärztliche Fachkraft oder durch eine interdisziplinäre Fachgruppe durchgeführt wurde [31]. Eine grundlegende Frage besteht allerdings darin, inwieweit komplexe Interventionen, wie sie u. a. die IMST darstellt, mit den üblichen Outcomeparametern erfasst und in ihrer Wirksamkeit bestätigt werden können oder ob es hierzu nicht der Entwicklung und Operationalisierung von Outcomeparametern zur Entwicklung eines Wirksamkeitsmodells der IMST bedarf [21]. Unabhängig von dieser noch unbeantworteten Fragestellung gilt dennoch die IMST als „Goldstandard“ in der Behandlung von Patient*innen mit chronischen Schmerzen [22].

Das hier vorgestellte Modell kann dazu führen, bisherige – oftmals strukturell zu begründende – Unzulänglichkeiten in der ambulanten schmerzmedizinischen Behandlung zu reduzieren. Durch die dargestellte ambulante Struktur kann eine Schmerzerkrankung früh erkannt und somit eine geeignete Behandlungsform initiiert werden. Gleichzeitig ist durch das obligatorische modifizierte Assessment bereits zu Therapiebeginn eine hohe diagnostische Qualität gewährleistet. Durch das darauffolgende adaptive Programm gelingt es, eine individualisierte Therapie bei gleichzeitiger Ressourcenschonung umzusetzen.

Ein grundlegendes Problem in der Versorgung von Patient*innen mit chronischen Schmerzen ist in der häufig fragmentierten Versorgungslandschaft zu sehen. Hinzu kommt der Mangel an schmerzmedizinisch qualifizierten Ärzt*innen, sodass die Versorgung der Betroffenen in der Regel ohne ausreichende Netzwerkbildung bei den Primärversorgern stattfindet. Auch wird gefordert, dass als eine Maßnahme zur Verbesserung der Versorgungssituation eine Anpassung der Gebührenordnung wünschenswert sei. Diese sollte sich jedoch nicht nur auf einzelne abrechenbare Leistungen beziehen, sondern durchaus auch offen sein, um Strukturen, die in ihrem Angebot die Komplexität chronischer Schmerzerkrankungen besser abbilden, zu unterstützen [27]. Hierzu zählt u. a. die Vergütung des IMA, ambulanter Komplexbehandlungen sowie die Vergütung von Patient*innenschulungen.

Auch wurde eine solche Struktur bisher nicht von den Fachgesellschaften beschrieben und in deren Klassifikationssystem berücksichtigt. Vielmehr besteht dort eine Lücke zwischen den sogenannten Zentren, die überwiegend an Kliniken angebunden sind, und den sogenannten Praxen bzw. Ambulanzen für Schmerzmedizin [25, 28]. Diese Versorgungslücke zwischen hoch spezialisierten und rein ambulanten Einrichtungen kann durchaus durch innovative Projekte, wie das hier Beschriebene, geschlossen werden. Der Vorteil der hier dargestellten Struktur liegt nicht nur in der fachlichen Zusammensetzung des Personals innerhalb einer Organisationsstruktur und dem damit vereinfachten Informationsaustausch. Ein vielleicht noch wesentlicherer Aspekt erscheint das adaptive Vorgehen zu sein. Hier kann die IMST in unterschiedlicher fachlicher und zeitlicher Intensität mit der verpflichtenden Durchführung des Reassessments und der damit optionalen (wiederholten) Anpassung der Therapieschwerpunkte verankert werden.

Unstrittig erscheint der Aspekt, dass in Abhängigkeit vom Chronifizierungsstadium unterschiedliche angepasste Therapieoptionen angeboten werden sollten. Dies setzt allerdings eine nachvollziehbare Steuerung von Fällen voraus. Hier kann sicherlich das IMA als adäquates Tool angesehen werden und sollte entsprechend in die Regelversorgung übernommen werden und flächendeckend Umsetzung finden [8].

## Fazit für die Praxis

Mit der hier vorgestellten adaptiven interdisziplinären multimodalen Schmerztherapieeinrichtung wird ein innovatives Projekt in der ambulanten Regelversorgung vorgestellt, dem es gelingt, eine Lücke zwischen hoch spezialisierten, meist klinischen Einrichtungen und der reinen ambulant-ärztlichen Versorgung zu schließen. Mit einem interdisziplinären und interprofessionellen Team, einer einheitlichen Arbeits‑, Dokumentations- und Interaktionsstruktur gelingt es, im ambulanten Setting komplexe Behandlungsprozesse mit einem hohen Flexibilitätsgrad umzusetzen. Dies ermöglicht individualisierte Behandlungsangebote und -konstellationen innerhalb der ambulanten Struktur, ohne aber aufgrund des adaptiven Charakters vorhandene Kapazitäten zu überlasten. Das vorgestellte Modell ist als eine Ergänzung zu den bisherigen etablierten (teil‑)stationären Behandlungssettings zu verstehen und kann diese keinesfalls ersetzen. Zudem muss die Wirksamkeit der adaptiven IMST evaluiert werden, um so den Stellenwert dieser Behandlungsform im Gesamtspektrum schmerzmedizinischer Behandlungspfade zu untermauern.

## Data Availability

Die dieser Publikation zugrundeliegenden Daten können bei berechtigtem Interesse über den korrespondierenden Autor angefragt werden.
